# Non-canonical NLRP3 inflammasome activation and IL-1β signaling are necessary to *L*. *amazonensis* control mediated by P2X7 receptor and leukotriene B_4_

**DOI:** 10.1371/journal.ppat.1007887

**Published:** 2019-06-24

**Authors:** Mariana M. Chaves, Debora A. Sinflorio, Maria Luiza Thorstenberg, Monique Daiane Andrade Martins, Aline Cristina Abreu Moreira-Souza, Thuany Prado Rangel, Claudia L. M. Silva, Maria Bellio, Claudio Canetti, Robson Coutinho-Silva

**Affiliations:** 1 Biophysics Institute Carlos Chagas Filho, Federal University of Rio de Janeiro, Rio de Janeiro/RJ, Brazil; 2 Institute of Biomedical Sciences, Federal University of Rio de Janeiro, Rio de Janeiro/RJ, Brazil; 3 Microbiology Institute Paulo de Goés, Federal University of Rio de Janeiro, Rio de Janeiro/RJ, Brazil; University of Florida, UNITED STATES

## Abstract

Leishmaniasis is a neglected tropical disease affecting millions of individuals worldwide. P2X7 receptor has been linked to the elimination of *Leishmania amazonensis*. Biological responses evoked by P2X7 receptor activation have been well-documented, including apoptosis, phagocytosis, cytokine release, such as IL-1β. It was demonstrated that NLRP3 inflammasome activation and IL-1β signaling participated in resistance against *L*. *amazonensis*. Furthermore, our group has shown that *L*. *amazonensis* elimination through P2X7 receptor activation depended on leukotriene B_4_ (LTB_4_) production and release. Therefore, we investigated whether *L*. *amazonensis* elimination by P2X7 receptor and LTB_4_ involved NLRP3 inflammasome activation and IL-1β signaling. We showed that macrophages from NLRP3^-/-^, ASC^-/-^, Casp-1/11^-/-^, gp91^phox-/- ,^ and IL-1R^-/-^ mice treated with ATP or LTB_4_ did not decrease parasitic load as was observed in WT mice. When ASC^-/-^ macrophages were treated with exogenous IL-1β, parasite killing was noted, however, we did not see parasitic load reduction in IL-1R^-/-^ macrophages. Similarly, macrophages from P2X7 receptor-deficient mice treated with IL-1β also showed decreased parasitic load. In addition, when we infected Casp-11^-/-^ macrophages, neither ATP nor LTB_4_ were able to reduce parasitic load, and Casp-11^-/-^ mice were more susceptible to *L*. *amazonensis* infection than were WT mice. Furthermore, P2X7^-/-^ *L*. *amazonensis-*infected mice locally treated with exogenous LTB_4_ showed resistance to infection, characterized by lower parasite load and smaller lesions compared to untreated P2X7^-/-^ mice. A similar observation was noted when infected P2X7^-/-^ mice were treated with IL-1β, i.e., lower parasite load and smaller lesions compared to P2X7^-/-^ mice. These data suggested that *L*. *amazonensis* elimination mediated by P2X7 receptor and LTB_4_ was dependent on non-canonical NLRP3 inflammasome activation, ROS production, and IL-1β signaling.

## Author summary

*Leishmania spp*. is a protozoan parasite that infects human and causes several diseases. *Leishmania amazonensis* causes cutaneous leishmaniasis (CL) and mucocutaneous leishmaniasis (MCL). *Leishmania* parasites preferentially infect macrophages. In macrophages, several mechanisms have been described as controlling *L*. *amazonensis* infection. Here, we showed that P2X7 receptor and LTB_4_ eliminated *L*. *amazonensis* in macrophages by a pathway dependent on non-canonical NLRP3 inflammasome activation and IL-1β signaling.

## Introduction

Leishmaniases are a group of neglected human infectious diseases that affect more than 12 million people worldwide, with 1.5 million of new cases per year [1,2]. The protozoan parasites of *Leishmania spp*. cause several clinical manifestations, from skin lesions (cutaneous leishmaniasis) to visceral injuries (visceral leishmaniasis) that may be fatal [3]. In the South America, *Leishmania amazonensis* is an important causative agent of Leishmaniasis.

*Leishmania* infect phagocytic cells in host mammalian cells, including macrophages. Ironically, these cells are responsible for parasite control upon membrane receptor activation via various effector mechanisms [4]. Among the several mediators that affect macrophage function, purinergic receptor activation has been described as important for *L*. *amazonensis* infection control [5,6]. Purinergic receptors are activated by extracellular nucleotides and are divided in two families: P2Y and P2X. P2Y receptors are metabotropic receptors coupled to G proteins, while P2X receptors are ionotropic receptors activated by extracellular ATP (eATP) [7]. The subtype P2X7 receptor was implicated in the control of several intracellular pathogens, including *T*. *gondii* [8–10], *Chlamydia spp*. [11,12] and *Mycobacterium tuberculosis* [13,14]. Our previous work reported that P2X7 receptor was important for *L*. *amazonensis* control by a mechanism dependent on leukotriene (LT) B_4_ [15].

Pathogen recognition by cells of the immune system occurs through a large number of extra and intracellular receptors. This process can lead to the synthesis of inflammatory lipid mediators, such as LTs [16]. LTs constitute a family of inflammatory mediators formed from arachidonic acid metabolism by 5-lipoxygenase (5-LO) [17]. Among 5-LO products, LTB_4_ is recognized as a pivotal neutrophil chemotactic factor. However, several reports also pointed to LTs as immunomodulators, participating in the control of infections by pathogens such as *Trypanosoma spp*. [18]. In addition, Serezani and collaborators [19] demonstrated LTB_4_ participation in *L*. *amazonensis* elimination. Furthermore, other studies have suggested the participation of LTs in the production of IL-1β-mediated inflammation by the NLRP3 inflammasome [20].

Stimulation of pattern-recognition receptors (PRRs) such as PAMPs and DAMPs (*pathogen- and danger-associated molecular patterns*, respectively) in the immune system were associated with an inflammatory cellular response that included the production of cytokines and chemokines [21]. One of the cellular systems activated by PRRs is the inflammasome platform, a cytoplasmic multiprotein complex that mediates IL-1β and IL-18 secretion [22,23]. The most well-characterized inflammasome is NLRP3. NLRP3 inflammasome activation may be accomplished by a wide variety of structurally varied agonists, including pathogenic organisms, pore-forming toxins, and DAMPs [24]. NLRP3 activation requires two signals, where P2X7 receptor is recognized as one of the major secondary signals for NLRP3 inflammasome activation [25,26]. Furthermore, NLRP3 inflammasome activation results in canonical or non-canonical activation: canonical NLRP3 inflammasomes convert pro-caspase-1 into active enzyme caspase-1 (Casp-1) [27], and the undefined non-canonical inflammasome promotes activation of pro-caspase-11 (Casp-11) [28,29]. The participation of the NLRP3 inflammasome in the elimination of *L*. *amazonensis* in a nitric oxide-dependent manner has already been demonstrated [30]. Also, a recent paper showed non-canonical NLRP3 inflammasome activation by lipophosphoglycan (LPG) from *Leishmania* membrane and casp-11 is important to the infection control [31].

Based on this rationale, it is reasonable to suggest that the P2X7 receptor, LTB_4_, and IL-1β may participate in the same pathway, leading to the control of *L*. *amazonensis*. Therefore, the objective of this study was to investigate the mechanisms of elimination of *L*. *amazonensis* when P2X7 receptor is activated by eATP.

## Results

### Control of *L*. *amazonensis* infection via P2X7 receptor depended on NLRP3 inflammasome activation and IL-1R signaling

A number of studies demonstrated the role of the pro-inflammatory cytokine IL-1β in protection against pathogens such as *Toxoplasma gondii* and *Trypanosoma cruzi* [32–34]. Therefore, we hypothesized that the P2X7 receptor may mediate the elimination of *L*. *amazonensis* in a manner dependent on NLRP3 inflammasome activation.

**Fig 1:**
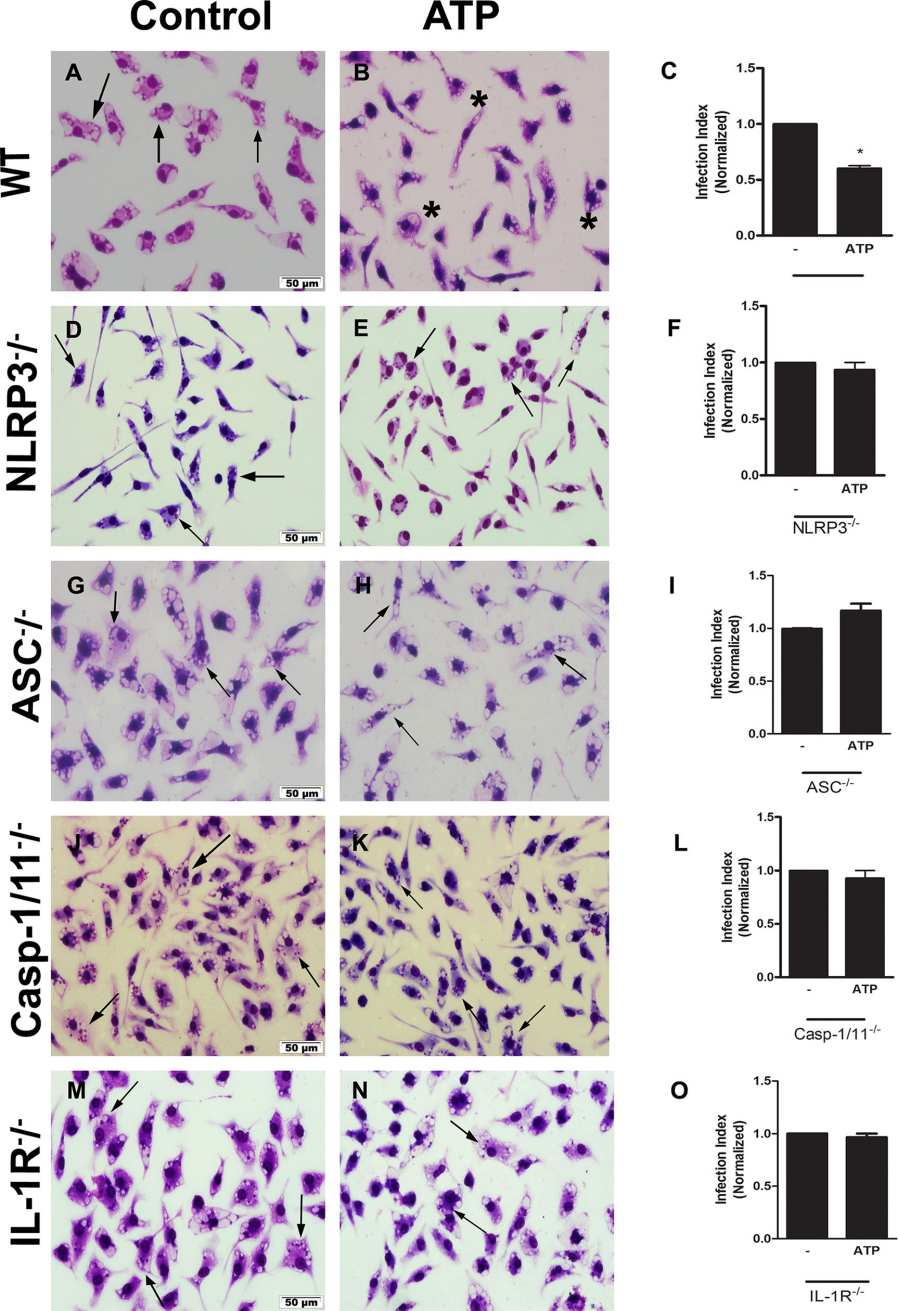
*L*. *amazonensis* control via P2X7 receptor was dependent on NLRP3 inflammasome and IL-1R signaling. Peritoneal macrophages from C57BL/6 (A-C), NLRP3^-/-^ (D-F), ASC^-/-^ (G-I), Casp-1/11^-/-^ (J-L), and IL-1R^-/-^ (M-O) mice were infected with *L*. *amazonensis* promastigotes at the ratio of 10:1 (Leishmania:macrophage). After 4 hours, the free parasites were washed and after 24 hours, infected cells were treated (B, E, H, K, and N) or not (A, D, G, J, and M) with 500 μM of ATP. Twenty-four hours later, cells were stained with May-Grunwald-Giemsa and the infection index was determined by direct counting under light microscopy. Normalized values represent means ± SEM of 3–4 independent experiments performed in triplicate. Arrows correspond to vacuoles with *L*. *amazonensis* and asterisks represent empty vacuoles. (*P < 0.05) compared to the control group (without treatment).

Our data demonstrated that control of *L*. *amazonensis* via ATP was dependent on the NLRP3 inflammasome, because ATP did not reduce parasite burden in infected macrophages from NLRP3^-/-^ mice (Fig 1D–1F), ASC^-/-^ mice (Fig 1G–1I), and Casp-1/11^-/-^ mice (Fig 1J–1L); however, it did reduce parasite burden in WT mice (Fig 1A–1C). These results suggested that the assembly of the NLRP3 inflammasome is an important mechanism in the elimination of parasites triggered by the P2X7 receptor. Furthermore, when we treated IL-1R^-/-^ infected macrophages with ATP, we did not observe reductions in parasitic load (Fig 1M–1O), demonstrating that IL-1R signaling was important to *L*. *amazonensis* control, mediated by the P2X7 receptor.

### *L*. *amazonensis* control via LTB_4_ depended on NLRP3 inflammasome activation and IL-1R signaling

**Fig 2:**
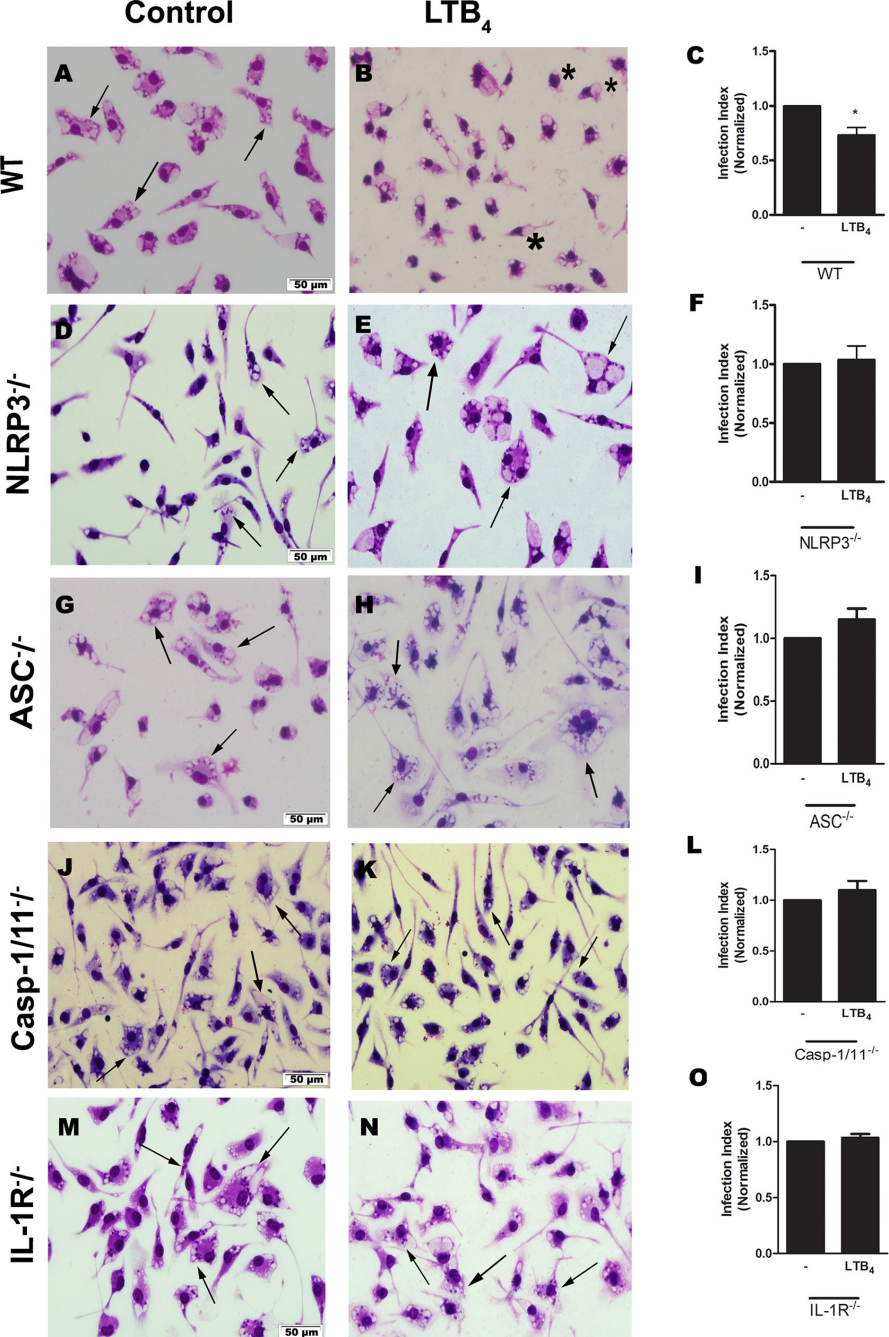
*L*. *amazonensis* control via LTB_4_ was dependent on the NLRP3 inflammasome and IL-1R signaling. Peritoneal macrophages from C57BL/6 (A-C), NLRP3^-/-^ (D-F), ASC^-/-^ (G-I), Casp-1/11^-/-^ (J-L), and IL-1R^-/-^ (M-O) mice were infected with *L*. *amazonensis* promastigotes at a ratio of 10:1 (Leishmania:macrophage). After 4 hours, the free parasites were washed and after 24 hours, infected cells were treated (B, E, H, K, and N) or not (A, D, G, J, and M) with 100 nM of LTB_4_. Twenty-four hours later, cells were stained with May-Grunwald-Giemsa and the infection index was determined by direct counting under light microscopy. Normalized values represent means ± SEM of 3–4 independent experiments performed in triplicate. Arrows correspond to vacuoles with *L*. *amazonensis* and asterisks represent empty vacuoles. (*P < 0.05) compared to the control group (without treatment).

We previously demonstrated that the elimination of *L*. *amazonensis* mediated by the P2X7 receptor depended on LTB_4_ production and release [15]. It had been demonstrated that LTB_4_ modulated activation of NLRP3-dependent inflammation following monosodium urate stimulation [20]. Therefore, using the same approach previously used, we tested whether LTB_4_ leishmanicidal activity was NLRP3 inflammasome-dependent. Indeed, the treatment of infected macrophages from NLRP3^-/-^ mice (Fig 2D–2F), ASC^-/-^ mice (Fig 2G–2I), Casp-1/11^-/-^ mice (Fig 2J–2L), and IL-1R^-/-^ mice (Fig 2M–2O) with LTB_4_ did not reduce parasite load; however, LTB_4_ reduced parasite load when infected macrophages from WT mice were treated (Fig 2A–2C), suggesting the importance of NLRP3 inflammasome and IL-1R signaling in elimination of *L*. *amazonensis* mediated by LTB_4_.

### *L*. *amazonensis* control via IL-1β depended on IL-1R signaling

**Fig 3:**
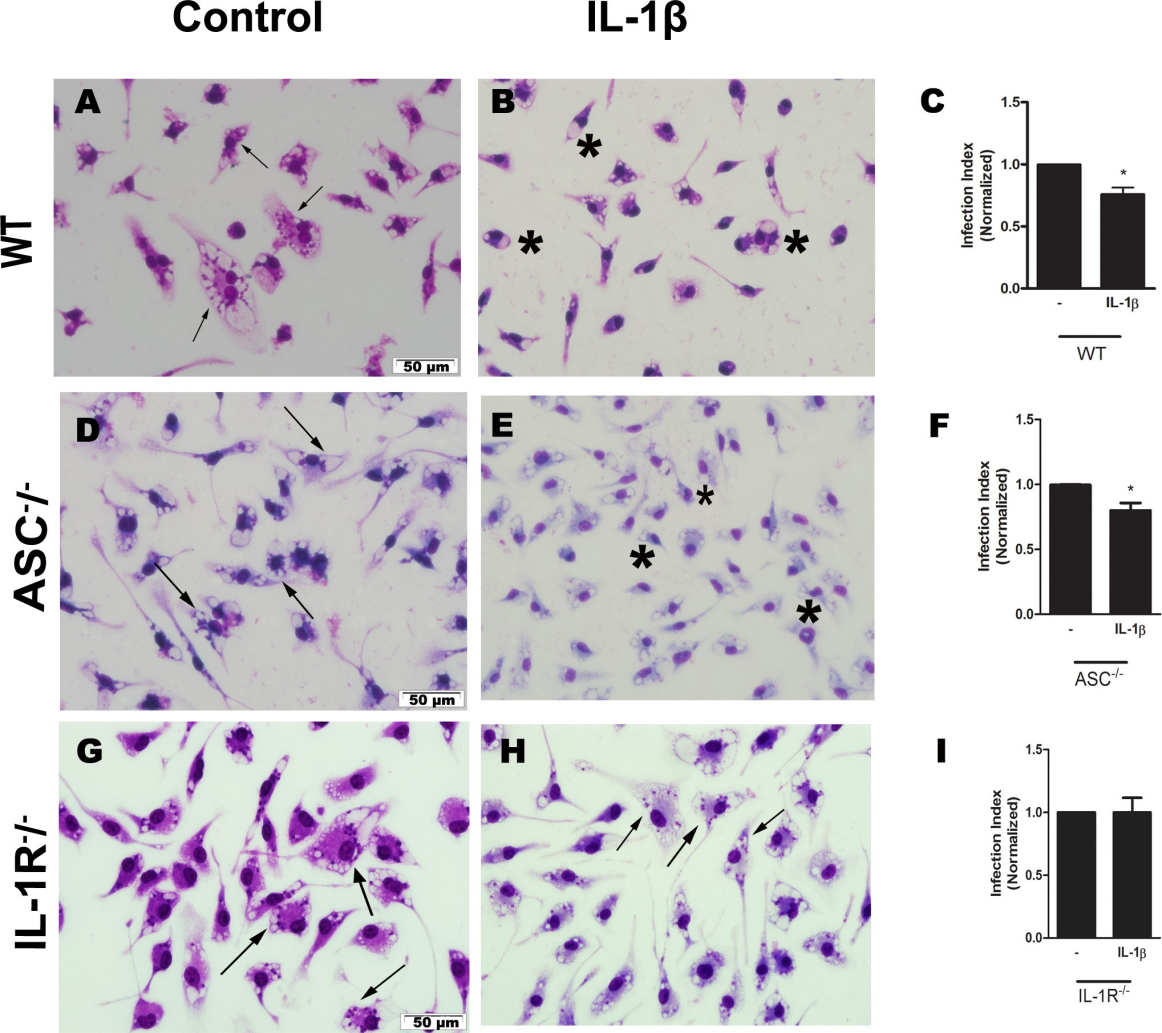
*L*. *amazonensis* control via IL-1β was dependent on IL-1R signaling. Peritoneal macrophages from C57Bl/6 (A-C), ASC^-/-^(D-F), and IL-1R^-/-^ (G-I) were infected with *L*. *amazonensis* promastigotes at the ratio of 10:1 (Leishmania:macrophage). After 4 hours, the free parasites were washed and after 24 hours, infected cells were treated with 100 pg/ml of IL-1β. Twenty-four hours later, cells were stained and the infection index was obtained. Normalized values represent means ± SEM of 3–4 independent experiments performed in triplicate. Arrows correspond to vacuoles with *L*. *amazonensis* and asterisks represent empty vacuoles. (*P < 0.05) compared to the control group (without treatment).

Infected macrophages from C57BL/6, ASC^-/-^, and IL-1R^-/-^ mice were treated with exogenous IL-1β and parasite load was determined. Infected cells from ASC^-/-^ mice (Fig 3D–3F) reduced parasite load following treatment with IL-1β, in similar fashion as IL-1β-treated WT cells. As expected, infected macrophages from IL-1R^-/-^ mice did not demonstrate an IL-1β effect (Fig 3G–3I).

To demonstrate the capacity of *L*. *amazonensis* to induce IL-1β release, we infected peritoneal macrophages with *Leishmania* and treated with or without ATP or LTB_4_. As seen in S1A Fig, only in infected macrophages was ATP or LTB_4_ able to induce IL-1β secretion. The infection by itself induced IL-1β secretion. However, when infected macrophages from Casp-11^-/-^ mice were stimulated with ATP or LTB_4_, we did not observe IL-1β release, suggesting that IL-β induced by P2X7 receptor and LTB_4_ during infection are dependent on Casp-11 (S1B Fig).

It is known that pannexin-1 is essential to Casp-11 activation mediated by P2X7 receptor [35]; therefore, we performed experiments blocking the pannexin-1 channel. We observed that neither ATP nor LTB_4_ reduced the parasitic load of infected macrophages when pannexin-1 inhibitor was added, suggesting that *L*. *amazonensis* control mediated by P2X7 receptor and LTB_4_ are dependent on pannexin-1 activation (S2 Fig).

### *L*. *amazonensis* control via P2X7 receptor and LTB_4_ depended on non-canonical NLRP3 inflammasome

**Fig 4:**
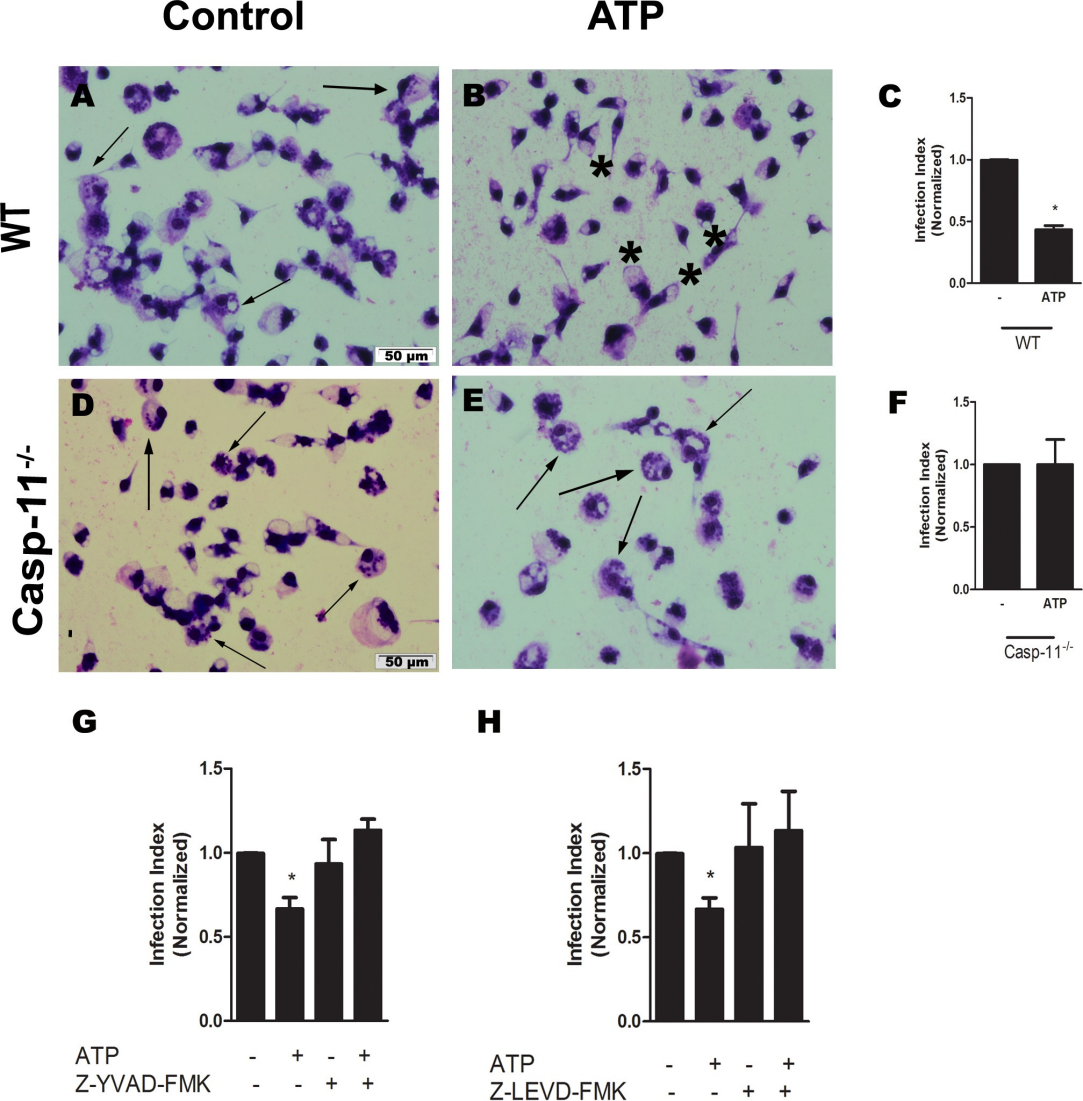
*L*. *amazonensis* control via ATP/P2X7 receptor was Casp-11 dependent. Peritoneal macrophages from C57Bl/6 (panels A-C, G, and H) and Casp-11^-/-^ (D-F) mice were infected with promastigotes of *L*. *amazonensis* at a ratio of 10:1 (Leishmania:macrophage). After 4 hours, the free parasites were washed and after 24 hours, infected cells were treated with ATP (500 μM; B and E). Infected macrophages were also treated with Z-YVAD-FMK and Z-LEVD-FMK, Casp-1 and Casp-11 inhibitors respectively, at the concentration of 2 μM 30 minutes before ATP treatment. Twenty-four hours later, the infection index was determined. Standard values represent means ± SEM of 3 independent experiments performed in triplicate. Arrows correspond to vacuoles with *L*. *amazonensis* and asterisks represent empty vacuoles. (*P < 0.05) compared to the control group (without treatment).

**Fig 5:**
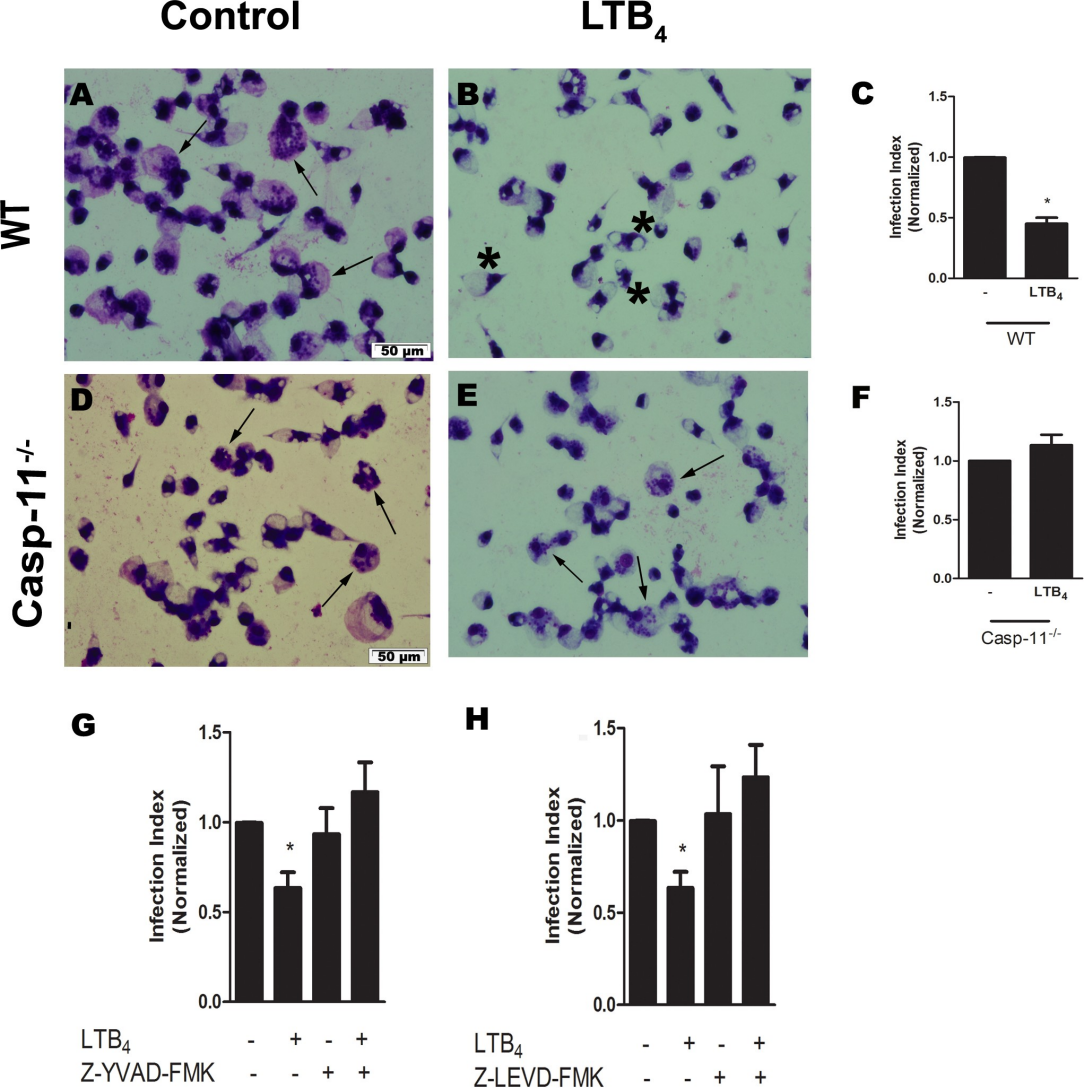
*L*. *amazonensis* control via LTB_4_ was Casp-11 dependent. Peritoneal macrophages from C57Bl/6 (panels A-C, G, and H) and Casp-11^-/-^ (D-F) mice were infected with promastigotes of *L*. *amazonensis* at a ratio of 10:1 (Leishmania:macrophage). After 4 hours, the free parasites were washed and after 24 hours infected cells were pretreated with 2 μM of Z-YVAD-FMK (G) or Z-LEVD-FMK (H) for 30 min. Subsequently, cells were treated with LTB_4_ (100 nM). Twenty-four hours later, the infection index was determined. Standard values represent means ± SEM of 3 independent experiments performed in triplicate. Arrows correspond to vacuoles with *L*. *amazonensis* and asterisks represent empty vacuoles. (*P < 0.05) compared to the control group (without treatment).

A previous study implicated Casp-11 in the elimination of intracellular pathogens [36]. Moreover, a recent work has showed that parasite membrane LPG from different species of Leishmania is able to activate casp-11 and consequent NLRP3 inflammasome in a non-canonical-dependent manner [31]. Therefore, we evaluated the importance of Casp-11 in the control of *L*. *amazonensis* mediated by P2X7 receptor and LTB_4_. We found that Casp-11 was essential for parasite burden reduction, because infected macrophages from Casp-11-deficient mice did not reduce parasite burden neither in the presence of ATP (Fig 4E and 4F) nor of LTB_4_ (Fig 5E and 5F). In addition, we used pharmacological inhibitors specific for Casp-1 and Casp-11, Z-YVAD-FMK and Z-LEVD-FMK, respectively, at concentrations of 2 μM prior to treatment with ATP (Fig 4G and 4H) or LTB_4_ (Fig 5G and 5H). Infected macrophages pre-treated with Z-YVAD-FMK and Z-LEVD-FMK did not reduce parasitic load after ATP exposure. These data suggest that both Casp-1 and Casp-11 are important for control of *L*. *amazonensis* via the P2X7 receptor and LTB_4_.

ROS is important for *Leishmania* control [37] as well as for non-canonical NLRP3 inflammasome activation; therefore, we performed experiments using peritoneal macrophages from gp91^phox-/-^ mice. Treatment of infected macrophages with ATP and LTB_4_ did not reduce parasitic load in macrophages from gp91^phox-/-^ mice, suggesting the importance of ROS in the P2X7 receptor-LTB_4_-NLRP3 activation axis during *L*. *amazonensis* infection (S3 Fig).

One of hallmarks of non-canonical inflammasome activation is the pyroptosis effect. We determined whether pyroptosis was involved in ATP and LTB_4_ anti-Leishmania effects. We found that ATP and LTB_4_ did not induce pyroptosis in infected macrophages (S4 Fig).

### Casp-11^-/-^ mice were susceptible to *L*. *amazonensis* infection

**Fig 6:**
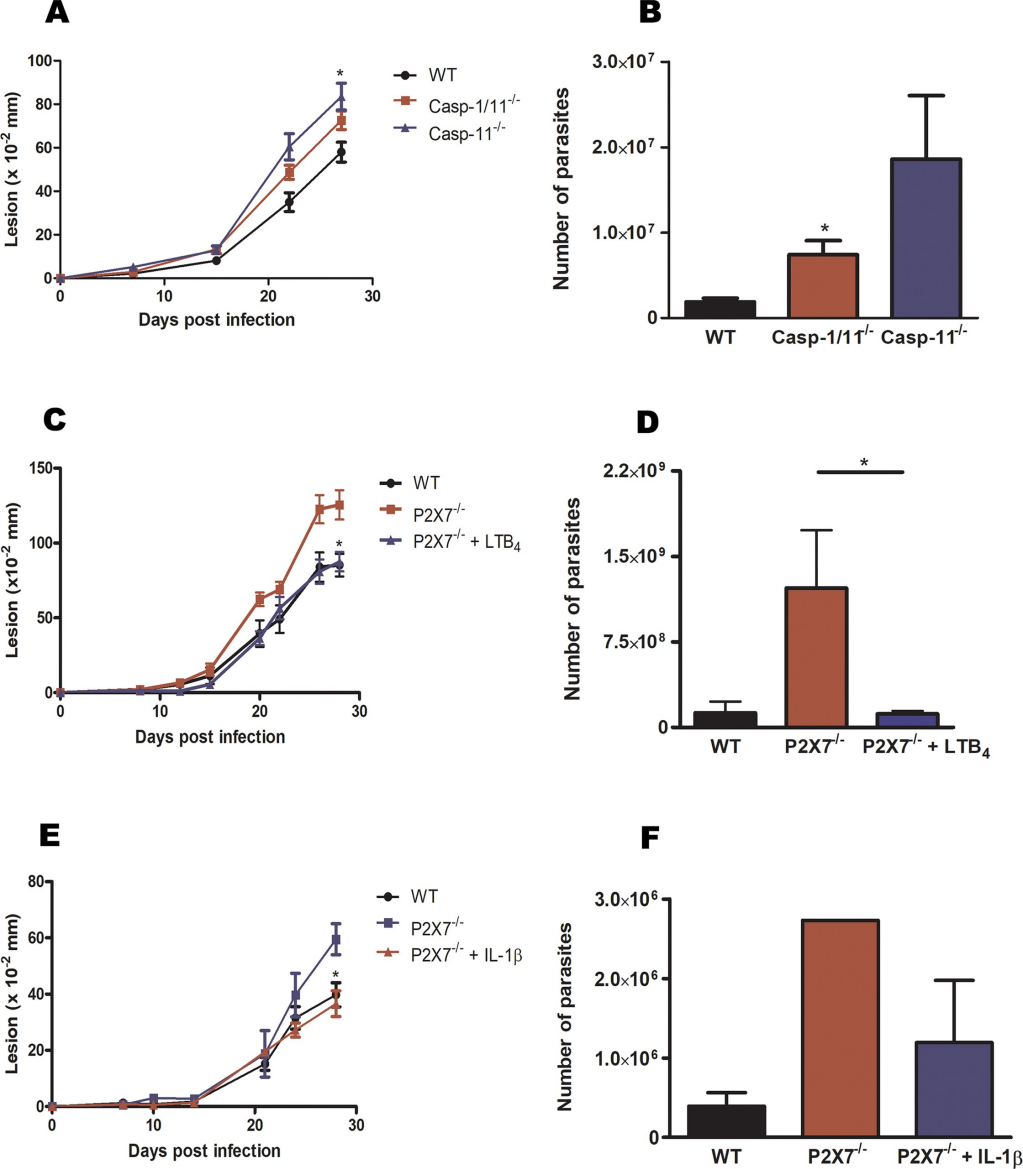
Casp-11^-/-^ mice were more susceptible infection and LTB_4_ and IL-1β restored resistance of P2X7 receptor^-/-^ mice to *L*. *amazonensis*. C57Bl/6 (A, B, C, D, E, F), P2X7^-/-^ (C, D, E, F), Casp-1/11^-/-^ (A, B), and Casp-11^-/-^ (A, B) mice were infected with 10^6^ promastigotes of *L*. *amazonensis* for 28 days and were treated with or without 5 ng of LTB_4_ (C, D) or 300 pg of IL-1β (E, F) twice weekly, for 3 weeks. Lesions was determined by thickening (A, C, E) and number of parasites by LDA as described (B, D, F). A, B correspond to mean ± SEM of a group of 9 animals; C, D correspond to mean ± SEM n = 8 mice in two independent experiments; E, F correspond to mean ± SEM of n = 5–4 animals (*P < 0.05).

To confirm the importance of Casp-11 during *L*. *amazonensis* infection, WT, Casp-1/11^-/-^, and Casp-11^-/-^ mice were infected in the footpad with *L*. *amazonensis*, and lesion development was followed for 28 days. As can be observed in Fig 6, Casp-11^-/-^ mice were susceptible to infection, presenting with larger lesions (6A) and larger parasitic loads than WT mice (6B). Casp-1/11^-/-^ mice also showed larger lesions and parasite loads compared to infected WT, agreeing with as study in which the importance of Casp-1/11 in resistance to *L*. *amazonensis* infection was demonstrated for the *L*. *amazonensis* PH8 strain [30].

### Exogenous LTB_4_ restored resistance in P2X7^-/-^ mice during *L*. *amazonensis* infection

Recent data from our group demonstrated that P2X7^-/-^ mice more susceptible to *L*. *amazonensis* infection than were WT mice [38]. We also demonstrated that macrophages from P2X7^-/-^ mice infected with *L*. *amazonensis* did not produce LTB_4_ after ATP stimulation [15]. Therefore, we hypothesized that the susceptibility of P2X7^-/-^ mice was due to ineffective LTB_4_ production. Therefore, when infected P2X7^-/-^ mice were treated with local LTB_4_ injections, we were able to restore resistance in these animals, demonstrated by smaller lesions and parasitic loads (6C, 6D) compared to those of vehicle-treated deficient mice (PBS). In addition, both lesion and parasite load were very similar to those of WT infected mice.

### Exogenous IL-1β restored resistance in P2X7^-/-^ mice during *L*. *amazonensis* infection

As previously mentioned, LTB_4_ induced IL-1β release. Therefore, we hypothesized that the susceptibility of P2X7^-/-^ mice to *L*. *amazonensis* infection could be due to deficient IL-1β production. To test this hypothesis, we treated infected P2X7^-/-^ mice with exogenous IL-1β and found that the treatment caused reduction of lesion size and parasite load to the same magnitude as was observed in WT mice (6E, 6F).

**Fig 7:**
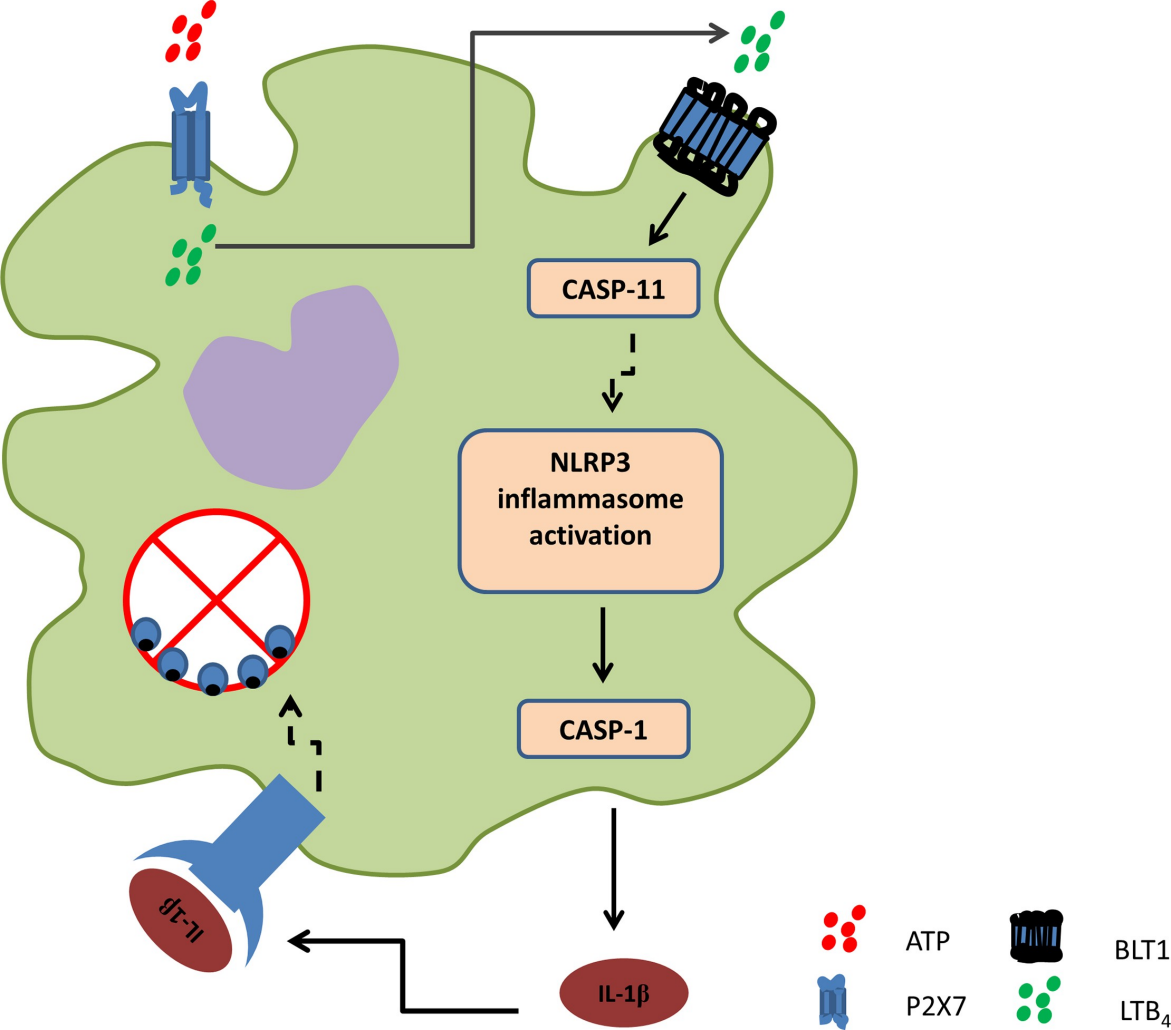
Proposed Mechanism. ATP activates the P2X7 receptor, which in turn leads to the production and release of LTB_4_. LTB_4,_ through a mechanism as yet unknown, activates Casp-11. Casp-11 activates the NLRP3 inflammasome, leading to maturation of Casp-1. Casp-1 cleaves pro-IL-1β into mature IL-1β as it is released into extracellular medium and activates IL-1 receptors. By a mechanism as yet unknown, activation of IL-1R leads to the control of *L*. *amazonensis* in macrophages.

Taken together, these data suggest that physiological ATP, through P2X7 activation, leads to LTB_4_ production and release. LTB_4_, per se, induced non-canonical NLRP3 inflammasome activation and IL-1β maturation, activating IL-1R to control *L*. *amazonensis* infection (Fig 7).

## Discussion

Previous data from our group showed that LTB_4_ was important to *L*. *amazonensis* control mediated by the P2X7 receptor [15]. However, the mechanism by which the P2X7 receptor together with LTB_4_ led to the elimination of *L*. *amazonensis* had not been elucidated. Purinergic receptors were important for elimination of several intracellular pathogens, including *T*. *gondii* [8–10], *Chlamydia spp*. [11,12] and *Mycobacterium tuberculosis* [13,14]. Furthermore, LTB_4_ was shown to be important in the control of *Histoplasma capsulatum* [39], *Klebsiella pneumoniae* [40], among other parasites. In the specific case of *L*. *amazonensis* infection, both P2X7 receptor and LTB_4_ were shown to be essential for parasite control [5,15,19,41]. The importance of the purinergic receptor-lipid mediator axis during leishmaniasis has been described [42]. In the present study, we demonstrated that ATP/P2X7 and LTB_4_ decreased *L*. *amazonensis* infection by a mechanism-dependent on non-canonical NLRP3 inflammasome activation, ROS production, and IL-1R signaling.

We showed that the NLRP3 inflammasome and IL-1R signaling were important for P2X7 and LTB_4_ receptor-dependent *L*. *amazonensis* elimination. Data in the literature point to the role of the NLRP3 inflammasome in *L*. *amazonensis* host resistance [30]. Furthermore, other pathogens also induced immune responses via inflammasome activation, including *T*. *gondii*, *T*. *cruzi* and *Paracoccidioides brasiliensis* [32–34,43]. However, in *L*. *major* infections, NLRP3 inflammasome activation appeared to be associated with infection susceptibility [44,45]. Therefore, activation of the NLRP3 inflammasome promoting resistance appeared to be species-specific in leishmaniasis. Our data suggested that NLRP3 inflammasome components, including NLRP3, ASC, and Casp-1/11 are important in *L*. *amazonensis* infection control, because, in the absence of these components, P2X7 activation and LTB_4_ did not decrease parasite load in infected macrophages.

The concept of P2X7 receptor activating the NLRP3 inflammasome is not new [46]. Other studies have also demonstrated the participation of LTB_4_ in NLRP3 inflammasome activation [20]. Moreover, a recent article demonstrated the importance of membrane permeabilization for IL-1β release [47]. Inhibition of IL-1β secretion in *L*. *donovani*-infected macrophages has been reported [48–50]. Furthermore, several species of *Leishmania* inhibited IL-1β production through GP63-dependent mechanisms [51]. Our previous study showed that macrophages infected with *L*. *amazonensis* released lower amounts of LTB_4_ when stimulated with ATP [15]. Therefore, it is reasonable to deduce that IL-1β and the NLRP3 inflammasome are important for the control of the infection caused by *Leishmania*, and also that the parasite has developed mechanisms to subvert the immune system, interfering with IL-1β synthesis/response.

Our data also suggested the participation of the non-canonical NLRP3 inflammasome in elimination of *L*. *amazonensis* mediated by the P2X7 receptor and LTB_4_, because, in the absence of Casp-11 or the use of a specific Casp-11 inhibitor, no reduction in the parasite load was detected after ATP or LTB_4_ treatment. Casp-11 was activated by LPS from Gram-negative bacteria [52,53]. Furthermore, other pathogens that do not express LPS, including *Candida albicans*, also led to Casp-11 activation [54]. Moreover, the importance of Casp-11 in control of enteric bacterial pathogens has been demonstrated [55]. It is also important to highlight that during the revision process of this manuscript, it was published an elegant study showing all hallmarks of caspase-11 activation in response to *Leishmania* infection, fact mediated by LPG molecule presented in parasite membrane [31]. Moreover, the same paper showed that non-canonical NLRP3 inflammasome activation is important to the control of Leishmania infection in vitro and in vivo. Recent work showed the importance of ROS for expression and activation of Casp-11 during infection [56]; another study showed that pyroptosis induced by Casp-11 activation was P2X7 receptor-dependent [35]. Moreover, the P2X7 receptor and LTB_4_ induced ROS release [57–62]. Furthermore, ROS is important to NLRP3 inflammasome assembly and control of the parasite during *Leishmania* infection [37]. Our data demonstrated that gp91^phox^, a NADPH oxidase component is important for *L*. *amazonensis* control mediated by P2X7 receptor and LTB_4_. However, caspase-11-dependent pyroptosis is not P2X7R-dependent because we did not observe pyroptosis 24 h after ATP treatment, suggesting that the parasite may inhibit this mechanism initially in order to evade host defense.

ATP is an intracellular molecule, however, it can actively and passively reach extracellular medium during inflammation [63]. Moreover, it is important to notice that in the immune response in vivo during *L*. *amazonensis* infection, several cells are recruited and infected that may even be a niche for replication of the parasite. The growth of Leishmania inside cells leads to rupture of these cells with consequent release of the intracellular contents to the extracellular medium. One of the molecules released after cell lysis is ATP, at millimolar intracellular concentrations. It is worth remembering that this happens simultaneously in several different cell types during in vivo infection, and that ATP is release actively during *L*. *amazonensis* phagocytosis by peritoneal macrophages [15]. Among the possible ways by which ATP is released to the extracellular compartment is through pannexin-1, a membrane channel/pore [64], and/or as a consequence of the death of infected cells could increase the extracellular ATP concentration, thereby controlling the infection through the activation of P2X7 receptors. Furthermore, Thorstenberg et al., 2018 [65] demonstrated in vivo release of ATP in lymph nodes during infection by *L*. *amazonensis* and showed that low doses of ATP (50 μM) decreased parasite burden in infected macrophage, in a pannexin-1- and P2X7 receptor-dependent mechanism. It is also worth mentioning that when released ATP is rapidly hydrolyzed by enzymes present in the host and parasite cell membranes, including CD39 and CD73.

We showed that lack of the Casp-11 enzyme caused susceptibility to *L*. *amazonensis* infection, because lesions and parasite loads were higher in these animals, as well as in Casp-1/11^-/-^ mice. Conversely, recent work from our group showed that during *T*. *gondii* control mediated by P2X7 receptor, Casp-11 was not important [66], suggesting that non-canonical NLRP3 inflammasome activation mediated by P2X7 receptor and LTB_4_ during infection is species-specific.

### Conclusions

Taken together, these results suggest that *L*. *amazonensis* control mediated by P2X7 receptor and LTB_4_ is dependent on production and release of IL-1β via non-canonical NLRP3 inflammasome activation. The understanding of this mechanism is of extreme importance for development of new therapeutic strategies in order to combat leishmaniasis.

## Materials and methods

### Ethics statement

All animal experiments were performed in accordance with Brazilian regulations conduct by Conselho Nacional de Experimentação Animal (CONCEA). All procedures using animals were approved by Comissão de Ética no Uso de Animais da Universidade Federal do Rio de Janeiro (CEUA-UFRJ) under number 077/15.

### Experimental animals

We used mice C57Bl/6, P2X7^-/-^ (Pfizer, USA), NLRP3^-/-^ (Genentech, USA), ASC^-/-^ (Genentech, USA), caspase (Casp)-1/11^-/-^ (Genentech, USA), Casp-11^-/-^ (Genentech, USA), and IL-1R^-/-^ (JAX Mice, USA) that were housed in a temperature-controlled room with a light/dark cycle and received food and water *ad libitum*. The P2X7 receptor^-/-^ and Casp-11^-/-^ mice were maintained at the Laboratory of Transgenic Animals (LAT) of the Institute of Biophysics Carlos Chagas Filho. The animals that were NLRP3^-/-^, ASC^-/-^, Casp-1/11^-/-^ were kindly provided by Dr. Dario Zamboni of the Medical School of USP-Ribeirão Preto, while the IL-1R^-/-^ animals were donated by Dr. Maria Bellio of the Institute of Microbiology Paulo de Góes of UFRJ. The mice used were of both genders, aged 6 to 16 weeks for the removal of peritoneal macrophages, and 6 to 8 weeks for in vivo experiments.

### Cell culture

All mice were euthanized in a CO_2_ chamber, followed by cervical dislocation as described in the report submitted and approved by the IBCCF ethics committee. Macrophages were obtained from the peritoneal cavity by inoculation and subsequent aspiration of 5 mL of cold PBS. The solution obtained was then centrifuged at 300 g for 10 minutes. Cells were counted by exclusion of dead cells using Trypan Blue (Sigma); 2 x 10^5^ cells per well were cultured with or without cover slips at 37°C and 5% CO_2_ for 1 hour. Non-adherent cells were then removed by washing twice with sterile PBS at 37°C. Macrophages were cultured in DMEM supplemented with 2 mM L-glutamine, penicillin (10 units/mL), streptomycin (10 μg/mL), and 10% inactivated fetal bovine serum.

### Parasites

Amastigote forms of *L*. *amazonensis* (MHOM/BR/75/Josefa) were obtained from popliteal lymph nodes of infected BALB/c mice for the maintenance of infectivity. Axenic promastigotes were transformed at 27°C into 199 medium supplemented with 2 mM L-glutamine, 10 units penicillin, 10 μg/ml streptomycin, 10% inactivated fetal bovine serum, 0.25% hemin, and 2% male sterile urine. Promastigotes were maintained until the tenth passage to maintain infective potential.

### *In vitro* infection

For macrophage infection, we used an MOI ratio of 10:1 (Leishmania:macrophage). The parasites were counted using a Neubauer chamber in an optical microscope. Infection was performed for 4 hours at 37°C and 5% CO_2_. After this time, the non-internalized parasites were removed by washing twice with PBS sterile. Infected macrophages were maintained in an incubator at 37°C and 5% CO_2_ for 24 hours.

### ATP, LTB_4_, and IL-1β treatment

The physiological agonist of P2X7 receptor, ATP (Sigma), LTB_4_ (Calbiochem), and IL-1β (R&D systems) were added at final concentrations of 500 μM, 100 nM, and 100 pg/mL, respectively. ATP and LTB_4_ were added for 30 min and IL-1β was added for 24 h.

### Infection index

The infection index was obtained by direct counting of infected cells under light microscopy. Cells were infected, and after 24 hours were stimulated with 500 μM eATP, 100 nM of LTB_4_ or 100 pg/mL of IL-1β. Twenty-four hours after the treatments, the infected macrophages, treated or not, were fixed and stained with a Panotico Fast kit (Laborclin) and mounted on slides for analysis by optical microscope. The infection index was determined from the infected macrophages count and also by the mean number of parasites per infected macrophage. This number was obtained by counting at least 100 cells in a total of five fields. The results were expressed as the infection index, which was the percentage of infected macrophages multiplied by the mean number of amastigotes per infected macrophage, divided by 100, as described previously. [67].

### *In vivo* infection

Mice were infected in the dermis of the right footpad by intradermal injection of 10^6^ parasites. The growth of the lesion was accompanied by measurement of the thickness of the infected paw compared to the uninfected paw. After 28 days, the animals were euthanized and their footpads were removed and macerated for parasite load determination by the limiting dilution test (LDA) [68]. Briefly, serial fourfold dilutions were performed in 96-well microtiter plates. After 7–14 days at 27°C, the presence or the absence of promastigotes in the wells was determined. The final titer was the last well in which it was possible to detect the presence of at least one parasite. In addition, C57Bl/6 and P2X7^-/-^ mice were infected and after 7 days. Deficient mice were locally treated with 300 pg of IL-1β or 5 ng of LTB_4_ twice a week for three weeks. Subsequently, the animals were euthanized and their paws were removed for parasitic load determination.

### Statistical analysis

Data were analyzed using the program GraphPad Prism 5.0 and the determination of the significance among the various experimental groups was performed by determining the mean and standard error of the mean from the student t test or ANOVA post-test Tukey (more than two groups). The results were considered statistically significant if *P* < 0.05.

For detailed experimental protocols used in supporting information figures, please refer to S1 Methods.

## Supporting information

S1 Methods Supporting information methods. (DOC)

S1 Fig LTB_4_ and P2X7 increase IL-1β production in a CASP-11^-/-^ dependent-manner. Peritoneal macrophages (2.0 x 10^5^) from WT (A) and CASP-11^-/-^ (B) mice were infected with stationary-phase *L*. *amazonensis* promastigotes for 1h. Quickly ATP and LTB were added in culture by 30 minutes. Following 4 h cells cultures were centrifuged by 10 minutes at 1200 RPM and supernantants were harvest to measured IL-1β by ELISA. Data correspond to the mean ± SEM values of n = 2 experiments performed in triplicate, with pooled cells from 4 to 5 animals.
(TIF)

S2 Fig ROS from NADPH-oxidase is involved in anti-amastigote response by ATP and LTB_4_. Peritoneal macrophages from WT (A) and gp91phox^-/-^ (B) were infected with *L*. *amazonensis*. Infected cells were treated by 30 minutes with ATP and LTB_4_ 24 h post infection. After, macrophages were fixed 30h post treatment, stained with panoptic, and the parasite load in infected macrophages was quantified as the “infection index” (% of infection x number of amastigote/total number of cells/100). Data correspond to the mean ± SEM values of n = 2 experiments performed in triplicate, with pooled cells from 4 to 5 animals.
(TIF)

S3 Fig Pannexin-1 is important to anti-parasitic effects by ATP and LTB_4_ treatment. Peritoneal macrophages from C57Bl/6 were infected with stationary-phase *L*. *amazonensis* promastigotes for 4h. Post 24 h infected cells were treated with Pannexin-1 antagonist CBX (50μM) for 30 minutes, following by stimulation with ATP and LTB_4_ for 30 minutes. Infected macrophages were fixed 30h post treatment, stained with panoptic, and the parasite load in infected macrophages was quantified as the “infection index” (% of infection x number of amastigote/total number of cells/100). Data correspond to the mean ± SEM values of n = 2 experiments performed in triplicate, with pooled cells from 4 to 5 animals.
(TIF)

S4 Fig Pyroptosis is not triggered after Casp-11 activation mediated by P2X7 receptor and LTB_4_ during L. amazonensis infection. Peritoneal macrophages from C57Bl/6 mice were infected with stationary-phase *L*. *amazonensis* promastigotes for 4h (MOI 10:1). Followed 24 h of *L*. *amazonensis* infection, the macrophages were treated or not with 500μM of ATP; or 100 nM of LTB4, during 30 minutes. As positive control, macrophages were treated with 0.1% triton X-100 in a cell culture media. The supernatant was collected after 24 h of treatment. The free lactate dehydrogenase (LDH) levels were measured using the LDH enzymatic Kit (Bioclin-BRA), according to the manufactured instructions). Data correspond to the mean ± SEM values of n = 2 experiments performed in triplicate, with pooled cells from 4 to 5 animals.
(TIF)
