# Supplementary material for: Non-canonical NLRP3 inflammasome activation and IL-1β signaling are necessary to L. amazonensis control mediated by P2X7 receptor and leukotriene B4
Source: PLoS Pathog. 2019 Jun 24;15(6):e1007887. doi: 10.1371/journal.ppat.1007887 (PMC6622556; doi:10.1371/journal.ppat.1007887)
Supplement: S1 Methods — (DOC) [file ppat.1007887.s001.doc]

**Supporting Information**

**Materials and methods**

*Infection index*

A total of 2.0 x 10^5^ peritoneal macrophages from C57Bl/6 and gp91phox^-/-^ mice were plated for 24 hours. Subsequently, cells were infected with stationary-phase *L. amazonensis* (MOI 10:1) for 4 hours. Twenty-four hours later, infected cells from C57Bl/6 mice were treated with or without pannexin-1 antagonist CBX (50 µM) for 30 minutes, followed by stimulation with ATP and LTB_4_ for 30 minutes and infected macrophages from C57Bl/6, and gp91phox^-/-^ mice were treated with ATP or LTB_4_ only. Subsequently, macrophages were fixed 30 h post treatment, stained with panoptic, and the parasite load in infected macrophages was quantified as the “infection index” (% of infection x number of amastigote/total number of cells/100).

*IL-1β production*

To measure IL-1β released in cell supernatants, peritoneal macrophages from WT, and CASP-11^-/-^ mice were plated in 96-well plates and infected with promastigotes of *L. amazonensis* (MOI 10:1) by 1 h. ATP (500 µM) and LTB_4_ (100 nM) were added by 30 minutes and removed. After 4 h, supernatants were collected and ELISA was performed.

*LDH assay*

Peritoneal macrophages were plated at 2 x 10^5^ cells in a 96-well plates. After 24 h, the macrophages were infected with *L. amazonensis*, (MOI 10:1) for four hours. After 24 h of *L. amazonensis* infection, the macrophages were treated with or without 500 µM ATP or 100 nM LTB4 for 30 minutes. As a positive control, macrophages were treated with 0.1% Triton X-100 in cell culture media. The supernatants were collected after 24 h of treatment. Free lactate dehydrogenase (LDH) levels were measured using an LDH enzymatic Kit (Bioclin-BRA), according to the manufactured instructions.
