# Supplementary figures and images for: Non-canonical NLRP3 inflammasome activation and IL-1β signaling are necessary to L. amazonensis control mediated by P2X7 receptor and leukotriene B4
Source: PLoS Pathog. 2019 Jun 24;15(6):e1007887. doi: 10.1371/journal.ppat.1007887 (PMC6622556; doi:10.1371/journal.ppat.1007887)

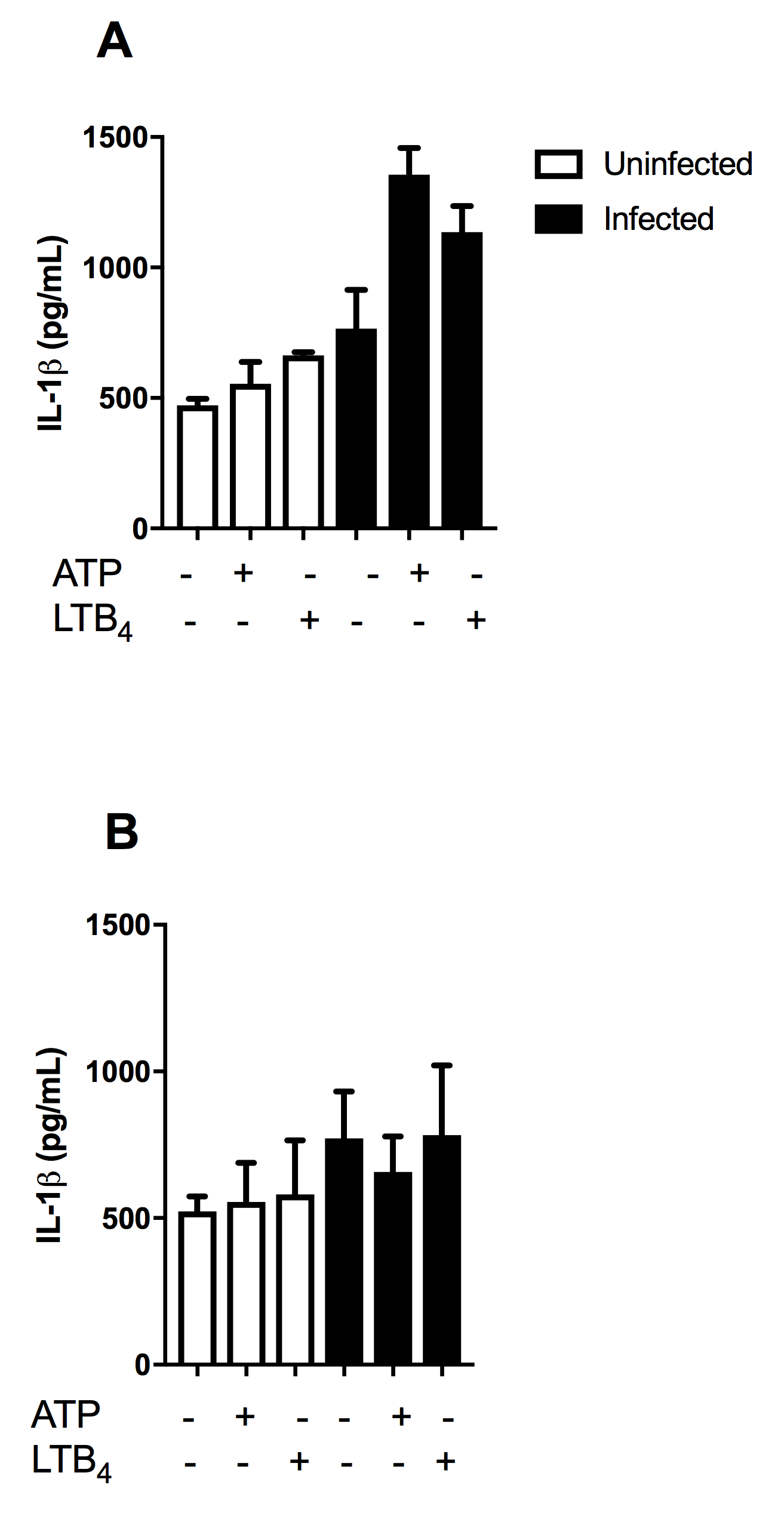

Supplement: S1 Fig — Peritoneal macrophages (2.0 x 10^5^) from WT (A) and CASP-11-/- (B) mice were infected with stationary-phase L. amazonensis promastigotes for 1h. Quickly ATP and LTB were added in culture by 30 minutes. Following 4 h cells cultures were centrifuged by 10 minutes at 1200 RPM and supernantants were harvest to measured IL-1β by ELISA. Data correspond to the mean ± SEM values of n = 2 experiments performed in triplicate, with pooled cells from 4 to 5 animals. (TIF) [file ppat.1007887.s002.tif]

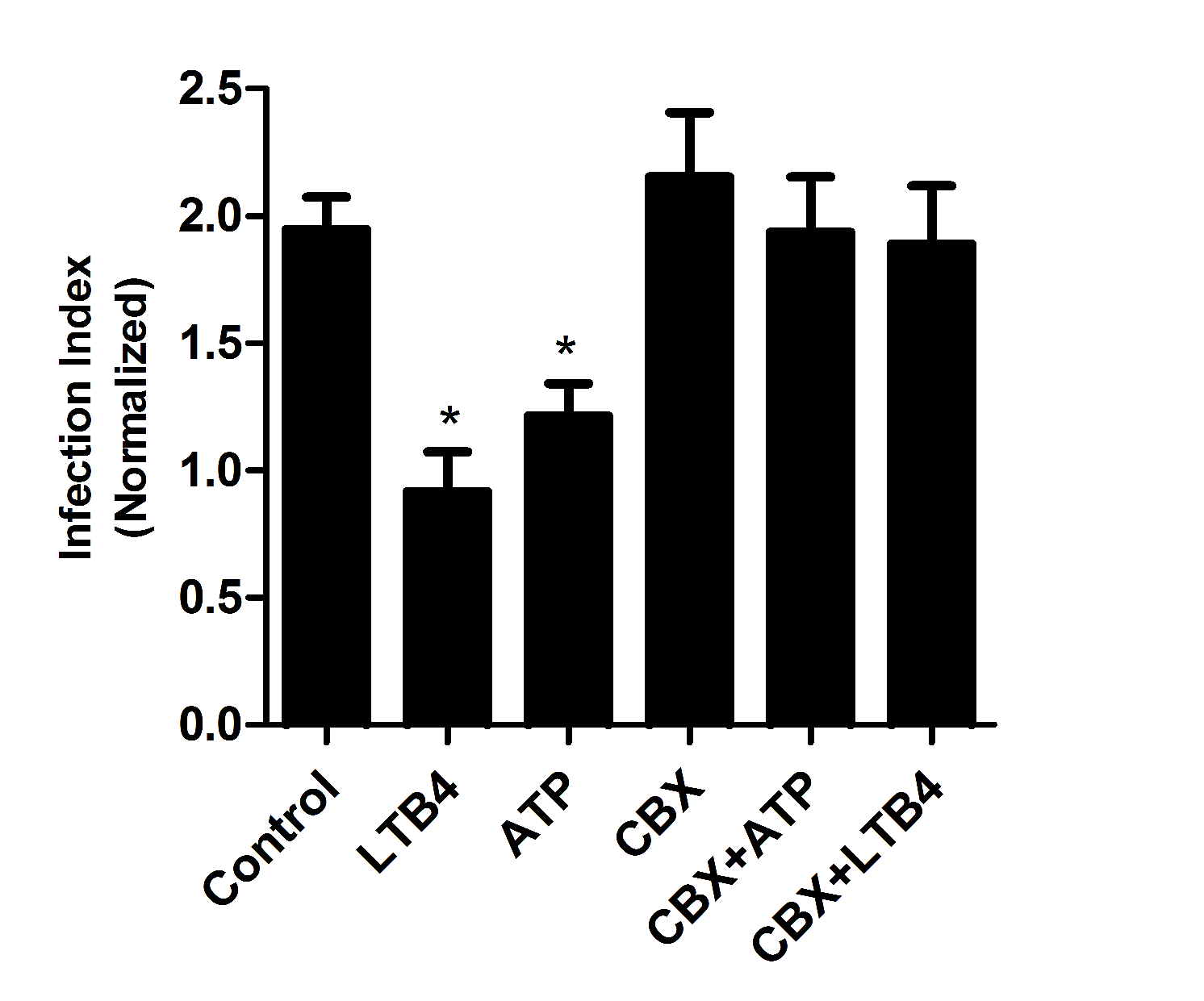

Supplement: S2 Fig — Peritoneal macrophages from WT (A) and gp91phox-/- (B) were infected with L. amazonensis. Infected cells were treated by 30 minutes with ATP and LTB4 24 h post infection. After, macrophages were fixed 30h post treatment, stained with panoptic, and the parasite load in infected macrophages was quantified as the “infection index” (% of infection x number of amastigote/total number of cells/100). Data correspond to the mean ± SEM values of n = 2 experiments performed in triplicate, with pooled cells from 4 to 5 animals. (TIF) [file ppat.1007887.s003.tif]

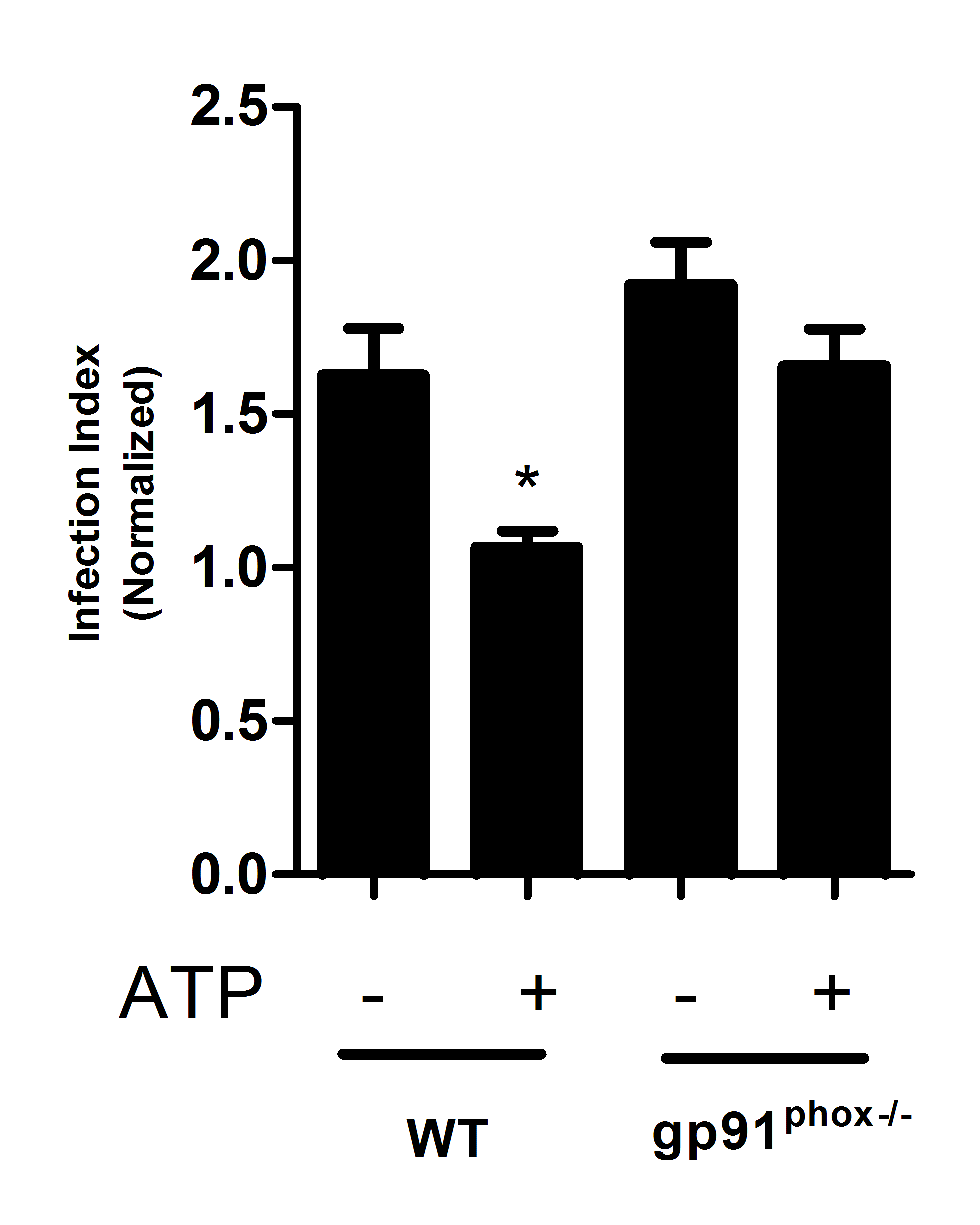

Supplement: S3 Fig — Peritoneal macrophages from C57Bl/6 were infected with stationary-phase L. amazonensis promastigotes for 4h. Post 24 h infected cells were treated with Pannexin-1 antagonist CBX (50μM) for 30 minutes, following by stimulation with ATP and LTB4 for 30 minutes. Infected macrophages were fixed 30h post treatment, stained with panoptic, and the parasite load in infected macrophages was quantified as the “infection index” (% of infection x number of amastigote/total number of cells/100). Data correspond to the mean ± SEM values of n = 2 experiments performed in triplicate, with pooled cells from 4 to 5 animals. (TIF) [file ppat.1007887.s004.tif]

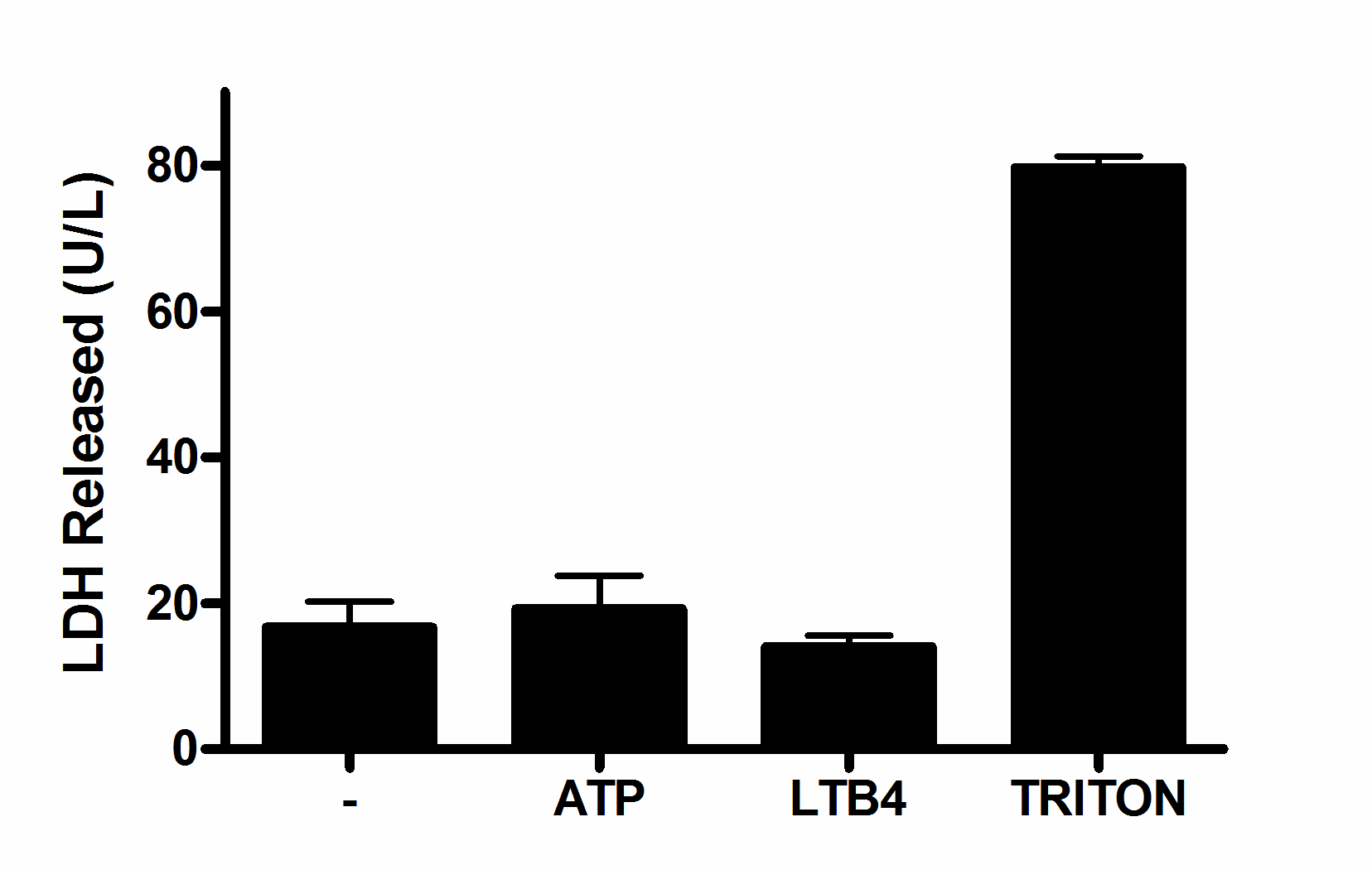

Supplement: S4 Fig — Peritoneal macrophages from C57Bl/6 mice were infected with stationary-phase L. amazonensis promastigotes for 4h (MOI 10:1). Followed 24 h of L. amazonensis infection, the macrophages were treated or not with 500μM of ATP; or 100 nM of LTB4, during 30 minutes. As positive control, macrophages were treated with 0.1% triton X-100 in a cell culture media. The supernatant was collected after 24 h of treatment. The free lactate dehydrogenase (LDH) levels were measured using the LDH enzymatic Kit (Bioclin-BRA), according to the manufactured instructions). Data correspond to the mean ± SEM values of n = 2 experiments performed in triplicate, with pooled cells from 4 to 5 animals. (TIF) [file ppat.1007887.s005.tif]
